# Preventive effect of intraoperative antiseptic irrigation on surgical site infections after deep brain stimulation: a retrospective cohort study

**DOI:** 10.3389/fneur.2026.1883788

**Published:** 2026-09-18

**Authors:** Baobin Liu, Chengyou Hong, Wei Ding, Mingyu Zhao, Zhijuan Chen, Kexin Li, Mengyao He, Yuchi Zhang, Junjie Gong, Jing Li, Chang Liu, Zengguang Wang

**Affiliations:** 1 Department of Neurosurgery, Tianjin Medical University General Hospital, Tianjin, China; 2 School of Medicine, Nankai University, Tianjin, China

**Keywords:** deep brain stimulation, hydrogen peroxide, infection control, povidone-iodine, surgical site infection

## Abstract

**Objective:**

Deep brain stimulation (DBS) is an effective treatment for movement disorders. However, hardware-related infections following DBS surgery represent a serious complication, with incidence rates ranging from 2.6% to 10%. DBS hardware-related infections can lead to significant clinical deterioration, including loss of therapeutic stimulation benefits, recurrence of initial symptoms, potential hardware removal, and increased need for anti-infective therapy. Nevertheless, the key risk factors and potential preventive measures for these infections remain unclear. This study aims to evaluate the efficacy of intraoperative prophylactic irrigation with hydrogen peroxide, 0.5% povidone-iodine solution, and their combination in reducing the rate of hardware infections following DBS surgery.

**Methods:**

A single-center retrospective cohort study was conducted, including patients who underwent DBS surgery between August 2016 and August 2024. Participants were categorized into one of four groups according to the intraoperative irrigation regimen used in clinical practice: saline, hydrogen peroxide, povidone-iodine, or combination. Only patients with complete operative and follow-up records were included. All patients were evaluated at four postoperative follow-up visits (1, 3, 6, and 12 months) to assess incision healing and surgical site infection (SSI) rates. Appropriate statistical analyses were employed to analyze variables of interest.

**Results:**

The overall surgical site infection (SSI) rate was 2.08% (8/385). The saline group exhibited a significantly higher infection rate compared to the intervention groups (4.2% vs. 1.03%, *p* = 0.039). The combination group did not demonstrate a synergistic effect (*p* > 0.05). Over approximately 8 years, 385 adult patients underwent 385 DBS procedures with bilateral electrode implantation, totaling 770 electrodes placed. Among these: Saline group: five SSI cases (5.26%) occurred within 1 year postoperatively. Pathogens identified: *Staphylococcus aureus* (*n* = 2) and *Staphylococcus epidermidis* (*n* = 3). Hydrogen peroxide group: 1 SSI case (1.02%) with *Staphylococcus epidermidis* identified. Povidone-iodine group: 1 SSI case (1.04%) with *Staphylococcus aureus* identified. Combination group: 1 SSI case with *Enterobacter hormaechei* identified.

**Conclusion:**

Although, the characteristics of most patients evaluated in this study and operative duration were not associated with the risk of SSI, our data indicate that wound irrigation with hydrogen peroxide or 0.5% povidone-iodine solution may contribute to reduced SSI risk following DBS implantation.

## 1 Introduction

Deep brain stimulation (DBS) is a safe and effective neuromodulation technique widely used for treating movement disorders, particularly demonstrating significant efficacy in Parkinson's disease, essential tremor, and dystonia. Subsequently, Benabid and his team refined DBS technology in the 1980s, successfully treating Parkinson's patients and establishing its pivotal role in modern functional neurosurgery (3).

Although DBS surgery is generally safe, hardware-related complications associated with implantable medical devices remain a major clinical concern (1). Foreign body implantation may trigger risks including infection, skin erosion, electrode migration, lead fracture/failure, and implantable pulse generator (IPG) malfunction (2). According to a systematic review and meta-analysis by Jitkritsadakul et al. (4) encompassing 28 studies (*n* = 5,114 patients), the overall hardware complication rate was 10.45%, with infection being the most frequent (5.12%), followed by electrode migration (1.60%), lead fracture (1.46%), IPG failure (1.06%), other component failures (0.73%), and non-infectious skin erosion (0.48%).

DBS-related infections can lead to severe consequences: patients typically require hospitalization, surgical debridement, long-term antibiotic therapy, and in severe cases, partial or complete hardware removal with staged reimplantation (5). A retrospective cost analysis by Chen et al. (6) revealed that the average total treatment cost for DBS infections in the United States exceeds $75,505, primarily attributed to reoperations (43.2%), inpatient care (31.8%), and antibiotic therapy (25.0%). These interventions not only increase physical and psychological burdens but also disrupt neuromodulation therapy, resulting in rebound motor symptoms (7).

To mitigate post-DBS infection risks, multiple preventive strategies have been explored: (1) Preoperative skin preparation with 70% ethanol-based antiseptic solutions (8); (2) Perioperative systemic antibiotic prophylaxis; (3) Intraoperative topical vancomycin powder application (9); (4) Antibiotic-impregnated envelopes (AIE) for IPG wrapping (10).

Among these, a randomized controlled trial by Halpern et al. (24) demonstrated that preoperative patient-applied ethanol-based solutions significantly reduced *Staphylococcus epidermidis* colonization (*p* < 0.01), through direct correlation with infection rates remains unconfirmed. A retrospective study by Abode-Iyamah et al. (2) reported reduced infection rates from 6.4% to 1.7% (*p* = 0.03) with local vancomycin powder. However, most current evidence remains limited to single-center observational studies, with scarce direct comparisons between prevention strategies.

Addressing this research gap, we designed a single-center retrospective cohort study to evaluate the impact of two topical antiseptics-−3% hydrogen peroxide and 0.5% povidone-iodine solutions—for intraoperative wound irrigation on post-DBS infection rates. This study pioneers the novel application of wound irrigation, an established infection prevention measure in orthopedic arthroplasty, to functional neurosurgery, aiming to provide high-level evidence for diversified DBS infection control (11).

## 2 Methods

### 2.1 Study design and patient categorization

We conducted a single-center retrospective cohort study, approved by the Institutional Review Board of Tianjin Medical University General Hospital (Approval No.: IRB2025-KY-447). Written informed consent was waived due to the retrospective, anonymized nature of the study.

The study included adult patients (≥18 years) with Parkinson's disease who underwent DBS surgery performed by Dr. Weidong Yang at Tianjin Medical University General Hospital between August 2016 and August 2024. Participants were stratified into four groups according to the intraoperative irrigation regimen used in clinical practice: (1) Saline group, (2) Hydrogen peroxide irrigation group, (3) Povidone-iodine irrigation group, (4) Combination irrigation group. The infusion plan is selected by the surgeon during the operation based on routine clinical practice, rather than being assigned according to a pre-determined research plan. Patients were not randomly assigned to any of the irrigation groups, and no randomization or allocation sequence was used. During the research period, there was no unified international guideline specifically stipulating the optimal intraoperative irrigation protocol for preventing infection in DBS surgery. In routine clinical practice, normal saline is usually used as the standard irrigation method, while hydrogen peroxide and povidone-iodine are also used as disinfection irrigation options. Additionally, in some cases, a sequential irrigation method of using hydrogen peroxide first and then povidone-iodine is adopted. The specific clinical factors influencing the choice of lavage regimens for individual patients were not predefined in the standardized protocols and were not systematically recorded in the medical records. According to the standard clinical procedures of our hospital, all DBS patients are required to undergo neuro-regulation adjustments and clinical evaluations at 1, 3, 6, and 12 months after the surgery. During these routine follow-ups, it is also required to assess the healing of the incision and surgical site infection (SSI). SSI was determined according to CDC criteria (13+) through comprehensive medical record review. Only patients with complete operative and follow-up records were included. Throughout the entire research period, all four irrigation methods were employed simultaneously, rather than being introduced one after another at different time points.

Inclusion criteria:

- Aged ≥ 18 years with clinically confirmed idiopathic Parkinson's disease.
- Scheduled for primary bilateral DBS lead implantation.
- Absence of active infection or immunosuppression preoperatively.

Exclusion criteria:

- Systemic antibiotic use within 30 days preceding surgery.
- Documented hypersensitivity to iodine compounds or hydrogen peroxide.
- Anticipated loss to follow-up or incomplete medical records.

### 2.2 Surgical procedure

All DBS procedures at our center were performed as single-stage implantations rather than staged surgeries. The regular surgical team consists of: a lead neurosurgeon, an assistant, a neurophysiologist, an anesthesiologist, a circulating nurse and an instrument nurse. All the operations were carried out under general anesthesia. Each case underwent multidisciplinary evaluation by a team comprising neurologists, neurosurgeons, and neuroradiologists to confirm diagnosis and select optimal targets: subthalamic nucleus (STN) or globus pallidus internus (GPi) for Parkinson's disease patients.

Electrode placement utilized MRI-based direct targeting coupled with frameless stereotaxy. Final lead positioning was confirmed through intraoperative electrophysiological verification:

- Microelectrode recording (MER) for GPi/STN targets.
- Macroelectrode stimulation for ventral intermediate nucleus (VIM).

The DBS hardware components, including the electrodes, extension cables and implantable pulse generator, are only opened from the sterile packaging after the target has been confirmed and before they are implanted immediately, in order to minimize air contamination.

### 2.3 Irrigation protocol

All procedures were performed by a consistent neurosurgical team in routine clinical practice, with intraoperative irrigation regimens applied according to clinical standards prior to wound closure:

1. Antibiotic prophylaxis
  Intravenous cefazolin (2 g) administered 1 h pre-incision. Penicillin-allergic patients received vancomycin (1 g).
2. Intraoperative irrigation (prior to wound closure)
  - Saline Group: 200 mL sterile 0.9% normal saline irrigation for 3 min.
  - H_2_O_2_ Group: 100 mL 3% hydrogen peroxide solution with 3-min dwell time, followed by 100 mL saline rinse.
  - PI Group: 100 mL 0.5% povidone-iodine solution with 3-min dwell time, followed by 100 mL saline rinse.
  - Combination Group: Sequential irrigation with 100 mL H_2_O_2_ (3-min dwell) → 100 mL PI (3-min dwell) → 100 mL saline neutralizing wash.

For all cases, after the incision was made, a conventional iodine-containing antibacterial surgical film was routinely applied to the skin around the incision to reduce bacterial contamination of the skin.

### 2.4 Management of SSI cases

Upon identification of surgical site infection (SSI), patients were immediately admitted for emergency management:

**1. Surgical intervention**
  - Radical local debridement.
  - Sequential irrigation protocol: 100 mL 3% H_2_O_2_ (3-min dwell) → 100 mL 0.5% povidone-iodine (3-min dwell) → 100 mL saline wash.
  - Hardware preservation with primary closure.

**2. Antimicrobial therapy**

The antimicrobial stewardship protocol, stratified by pathogen profile and drug susceptibility, was implemented as schematized below (Figure 1) with evidence-based justification.

**3. Concomitant structured surveillance was implemented per the following protocol:**

**Figure 1:**
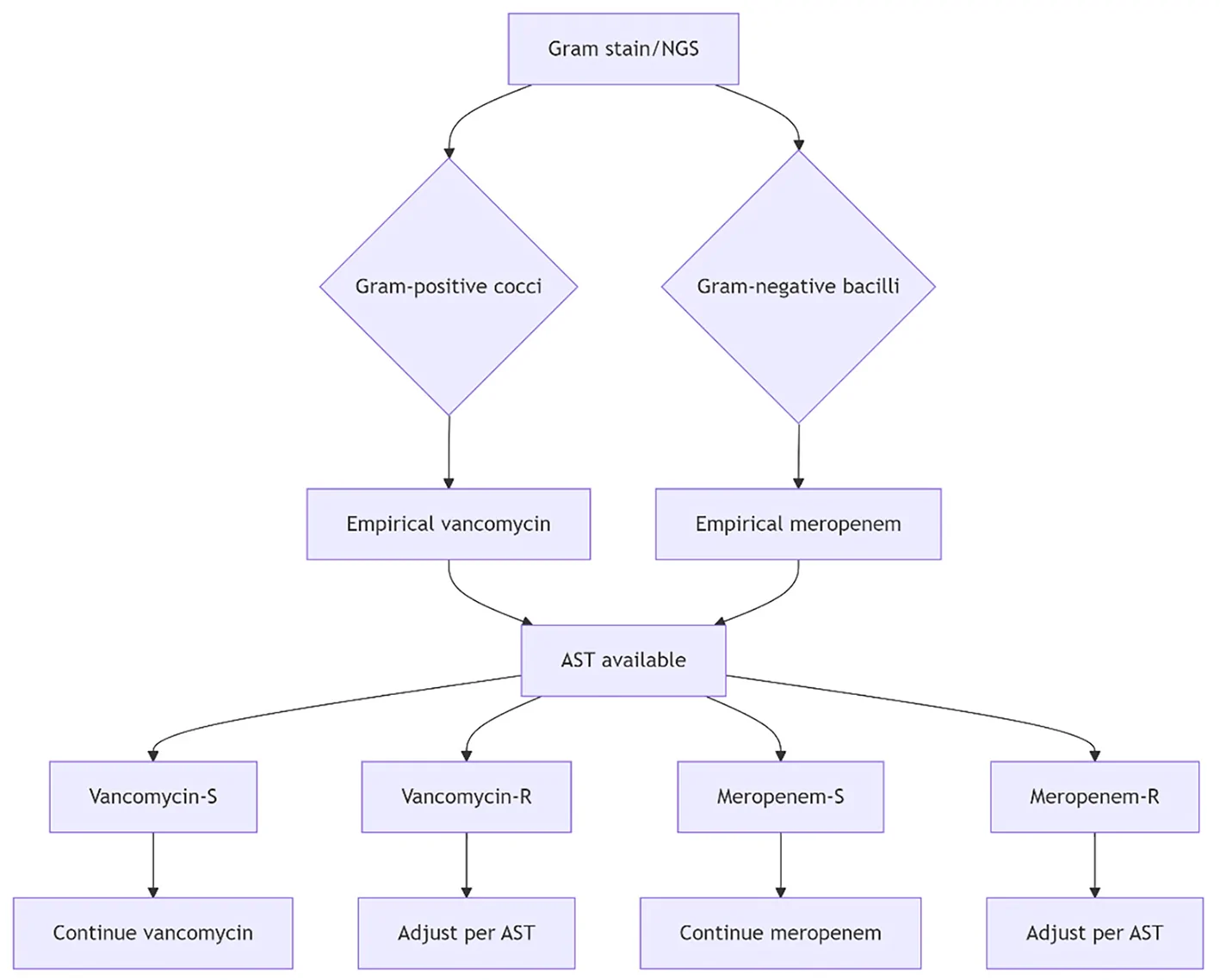
Flowchart of the antibacterial drug management plan based on drug sensitivity results and pathogen stratification. This figure presents a clinical decision-making path based on evidence-based evidence. Firstly, based on the Gram-positive cocci or Gram-negative bacilli indicated by Gram staining or next-generation sequencing (NGS) results, meropenem or vancomycin are respectively selected for empirical treatment. Subsequently, based on the availability of the results of the antimicrobial susceptibility test (AST) and the specific drug susceptibility report, the antimicrobial regimen was reviewed and optimized to achieve targeted treatment and the reduction of the antimicrobial drug ladder. S, sensitive; R, drug-resistant.

Phase Frequency Assessments

IV Therapy Daily Temperature, wound drainage

IV Therapy Q72h CRP, PCT, CBC, LFTs/RFTs

Oral Therapy Weekly Temperature, wound healing, CRP/PCT/CBC/LFTs/RFTs

**4. Treatment duration and discontinuation**
  - Sutures removed at postoperative day 7 based on healing
  - Antibiotics discontinued after 4 weeks total (IV + oral) when: Afebrile >72 h; Normal inflammatory markers (CRP/PCT); Normal wound examination
  - Monthly surveillance for 12 months post-cessation

**5. Treatment failure management**

Persistent infection after second debridement mandated: complete removal of affected hardware; Staged reimplantation after infection eradication.

Follow-up and endpoint definitions

Patients underwent routine postoperative clinical follow-up at 1, 3, 6, and 12 months after surgery (12). Surgical site infection (SSI) was diagnosed according to CDC criteria (8). Microbiological confirmation was performed using next-generation sequencing (NGS).

## 3 Results

A total of 385 patients were enrolled (214 males, 171 females), with mean age 62.5 ± 8.8 years, fasting blood glucose 5.6 ± 1.2 mmol/L, and BMI 24.9 ± 3.1 kg/m^2^. ASA class II patients accounted for 86.7% of the cohort, while males constituted 55.6%. Mean operative duration was 211 ± 32 min. No significant differences existed in demographic characteristics or surgical parameters among the four groups (*P* > 0.05, Table 1).

**Table 1:** Comparison of baseline characteristics among four groups.

Variable	Saline group (*n* = 95)	H_2_O_2_ group (*n* = 96)	Iodine group (*n* = 97)	Combination group (*n* = 97)	*P*-value
Male, *n* (%)	53 (55.8%)	54 (56.3%)	54 (55.7%)	53 (54.6%)	0.998
Age (years)	62.8 ± 9.0	62.2 ± 8.5	62.6 ± 9.0	62.4 ± 8.7	0.947
BMI (kg/m^2^)	24.8 ± 3.2	25.0 ± 3.0	24.9 ± 3.1	25.0 ± 3.0	0.984
Fasting glucose (mmol/L)	5.7 ± 1.3	5.5 ± 1.1	5.6 ± 1.2	5.6 ± 1.2	0.892
ASA grade (I/II/III)	32/48/15	35/46/15	33/49/15	34/47/16	0.999
Operative time (min)	210 ± 30	212 ± 34	210 ± 33	212 ± 31	0.956

1. Data presentation: categorical variables (Sex, ASA grade) expressed as counts/percentages or grade distributions; Continuous variables reported as mean ± standard deviation.

2. Statistical tests: sex distribution: Chi-square test (χ^2^ = 0.15); Age/BMI/operative time: one-way ANOVA (F < 0.08 for all); fasting glucose: Kruskal–Wallis test (H = 0.52; used due to mild non-normality per Shapiro–Wilk test); ASA grades: Chi-square test (χ^2^ = 0.35).

3. Interpretation: All P-values > 0.05 indicate no statistically significant differences between groups.

All patients underwent successful suture removal at postoperative days 7–8 without irrigation-related adverse events (e.g., tissue necrosis, allergic reactions) in the intervention groups. No wound healing complications or gas embolism occurred. Among the 385 patients completing 1-year follow-up, 8 SSI cases were documented (overall rate: 1.82%). Infection distribution revealed:

- Saline group: five cases (5.26%).
- Hydrogen peroxide group: one case (1.02%).
- Povidone-iodine group: one case (1.04%).
- Combination group: one case (1.03%).

Although each intervention group demonstrated ≈80% lower infection rates than controls, statistical significance was not achieved due to limited sample size per group and low event frequency (*P* > 0.05). Pooled analysis of all intervention groups (*n* = 290) confirmed significant risk reduction vs. controls (*P* = 0.039; Table 2). The combination regimen showed no synergistic effect compared to single-agent interventions (*P* > 0.05).

**Table 2:** Comparison of surgical site infection (SSI) by group.

Group	Patients	SSI events	Infection rate (%)	*p*-value vs. control
Saline	95	5	5.26%	— (Reference)
Hydrogen peroxide	96	1	1.04%	0.212
Povidone-iodine	97	1	1.03%	0.205
Combination	97	1	1.03%	0.205
All intervention groups	290	3	1.03%	0.039

(1) p-value calculation: Fisher's exact test (two-sided) for each intervention vs. Control. (2) Significance threshold: p < 0.05 considered statistically significant. (3) Synergistic effect: Combination vs. single interventions (hydrogen peroxide + povidone-iodine pooled): p = 1.000 (no significant difference). (4) Group Definitions: Saline irrigation; H_2_O_2_: Hydrogen peroxide irrigation; Iodophor: Povidone-iodine irrigation; Combination: H_2_O_2_ + Iodophor irrigation.

To further clarify the characteristics of these infections, we analyzed their anatomical distribution and depth. The data showed that among the eight cases of infection, three occurred on the chest wall (related to the IPG pouch), three were located on the head (at the electrode implant site), and two were in the posterior auricular region (Supplementary Table 1). In terms of depth, all the identified infections were classified as deep incisional SSI related to surgical instruments or organ/space SSI (such as the pouch or electrode pathway), and no superficial infections limited to the skin surface were reported. There was no significant difference in the distribution of infection sites between the saline group and the intervention group.

Microbiological analysis revealed *Staphylococcus aureus* in three cases (two in the saline group vs. one in the povidone-iodine group), *Staphylococcus epidermidis* in four cases (three in the saline group vs. one in the hydrogen peroxide group), and *Enterobacter hormaechei* in one case (in the combination group). Gram-positive cocci were identified in seven cases, and Gram-negative bacilli in one case. The distribution of pathogens did not demonstrate clinically significant differences between the groups, as detailed in Table 3.

**Table 3:** Pathogen distribution of surgical site infections (SSI) in DBS surgery by intervention group.

Pathogen	Saline group (*n* = 95)	H_2_O_2_ group (*n* = 96)	Iodophor group (*n* = 97)	Combination group (*n* = 97)	Total cases [*n* = 8, *n* (%)]
*Staphylococcus aureus*	2	0	1	0	3 (37.5%)
*Staphylococcus epidermidis*	3	1	0	0	4 (50.0%)
*Enterobacter hormaechei*	0	0	0	1	1 (12.5%)
Total SSI per group	5	1	1	1	8 (100%)
SSI rate per group	5.26%	1.04%	1.03%	1.03%	2.08%

(1) Group definitions: saline irrigation; H_2_O_2_: Hydrogen peroxide irrigation; Iodophor: Povidone-iodine irrigation; Combination: H_2_O_2_ + Iodophor irrigation. (2) Pathogen Significance: Staphylococci (S. aureus + S. epidermidis) dominated infections (87.5%), aligning with implant-associated infection epidemiology; Enterobacter hormaechei (gram-negative) appeared only in the combination group. (3) Statistical limitations: No significant pathogen distribution differences between groups (P > 0.99, Fisher's exact test) due to: (1) Small SSI sample size (n = 8); (2) Low event frequency in intervention groups (1 infection/group).

The median time to surgical site infection (SSI) onset was 28 days in the saline group, 29 days in the povidone-iodine group, 32 days in the hydrogen peroxide group, and 353 days in the combination group. In the combination group, one case of notably delayed infection was observed, with a latency of 353 days. In contrast, median times among the hydrogen peroxide, povidone-iodine, and saline groups were similar (32 vs. 29 vs. 28 days, respectively), showing no clinically significant differences, as presented in Table 4.

**Table 4:** Surgical site infection (SSI) onset time after DBS surgery.

Group	Patients (*n*)	SSI cases (*n*)	Onset time (days)	Mean time (days)	Median (days)	Time range (days)
Saline	95	5	23, 26, 28, 31, 35	28.6	28.0	23–35
H_2_O_2_	96	1	32	32.0	32.0	32
Iodine	97	1	29	29.0	29.0	29
Combination	97	1	353^*^	353.0	353.0	353.0

(1) Statistical limitation: No inferential analysis possible due to low SSI incidence in intervention groups (n = 1 per group). (2) Group Definitions: Saline irrigation; H_2_O_2_: Hydrogen peroxide irrigation; Iodophor: Povidone-iodine irrigation; Combination: H_2_O_2_ + Iodophor irrigation. (3) 353^*^Significant outlier (delayed onset).

Among the eight SSI cases in this study: during readmission for debridement and anti-infective treatment, none of the patients exhibited fever. All eight patients underwent uneventful suture removal on postoperative day eight following debridement, with no observed wound effusion or exudate during the observation period. Procalcitonin (PCT) and white blood cell count (WBC) showed no significant elevation throughout monitoring. C-reactive protein (CRP) levels were mildly elevated preoperatively upon admission but demonstrated a consistent downward trend postoperatively, returning to normal between postoperative days 6 and 9. Liver and kidney function tests revealed no clinically significant abnormalities during the monitoring period (see Table 5).

**Table 5:** Dynamic changes of laboratory parameters.

Patient ID	Parameter	At admission (Pre-op)	Post-op day 3	Post-op day 6	At discharge (Post-op Days 9-11)
1	CRP	8 mg/L	15 mg/L	5 mg/L	2 mg/L
	PCT	0.08 ng/ml	0.07 ng/ml	0.09 ng/ml	0.06 ng/ml
	WBC	8.5 × 10^9^/L	8.7 × 10^9^/L	8.3 × 10^9^/L	8.4 × 10^9^/L
2	CRP	9 mg/L	18 mg/L	8 mg/L	3 mg/L
	PCT	0.07 ng/ml	0.06 ng/ml	0.08 ng/ml	0.05 ng/ml
	WBC	9.0 × 10^9^/L	9.2 × 10^9^/L	8.8 × 10^9^/L	9.0 × 10^9^/L
3	CRP	38 mg/L	20 mg/L	10 mg/L	3 mg/L
	PCT	0.09 ng/ml	0.08 ng/ml	0.07 ng/ml	0.06 ng/ml
	WBC	9.2 × 10^9^/L	9.4 × 10^9^/L	9.1 × 10^9^/L	9.3 × 10^9^/L
4	CRP	42 mg/L	25 mg/L	12 mg/L	4 mg/L
	PCT	0.06 ng/ml	0.05 ng/ml	0.07 ng/ml	0.04 ng/ml
	WBC	8.8 × 10^9^/L	9.0 × 10^9^/L	8.6 × 10^9^/L	8.7 × 10^9^/L
5	CRP	7 mg/L	12 mg/L	6 mg/L	2 mg/L
	PCT	0.05 ng/ml	0.06 ng/ml	0.04 ng/ml	0.03 ng/ml
	WBC	8.2 × 10^9^/L	8.4 × 10^9^/L	8.1 × 10^9^/L	8.0 × 10^9^/L
6	CRP	40 mg/L	22 mg/L	11 mg/L	3 mg/L
	PCT	0.08 ng/ml	0.07 ng/ml	0.09 ng/ml	0.06 ng/ml
	WBC	9.5 × 10^9^/L	9.6 × 10^9^/L	9.2 × 10^9^/L	9.3 × 10^9^/L
7	CRP	37 mg/L	19 mg/L	9 mg/L	3 mg/L
	PCT	0.07 ng/ml	0.06 ng/ml	0.08 ng/ml	0.05 ng/ml
	WBC	9.1 × 10^9^/L	9.3 × 10^9^/L	8.9 × 10^9^L	9.0 × 10^9^/L
8	CRP	45 mg/L	28 mg/L	15 mg/L	5 mg/L
	PCT	0.09 ng/ml	0.08 ng/ml	0.10 ng/ml	0.07 ng/ml
	WBC	9.6 × 10^9^/L	9.7 × 10^9^/L	9.3 × 10^9^/L	9.4 × 10^9^/L

Antibiotic management: pathogen identification via Next-Generation Sequencing (NGS) indicated Gram-positive cocci in seven SSI cases, prompting empirical vancomycin administration. Antimicrobial susceptibility testing (AST) confirmed vancomycin susceptibility in all cases. The remaining case involved a Gram-negative *bacillus*, for which meropenem was administered empirically; AST subsequently confirmed meropenem susceptibility. Consequently, the initial intravenous antibiotic regimen was maintained for all eight patients without alteration. Following normalization of blood counts, CRP, and PCT levels, therapy was switched to oral antibiotics (selected based on AST results). The duration of intravenous antibiotic therapy ranged from 6 to 9 days, and the hospital length of stay ranged from 9 to 11 days (see Table 6).

**Table 6:** SSI cases—pathogen profile and antibiotic management.

Patient ID	Pathogen type	Identified bacteria	Intravenous antibiotic	Duration of IV (days)	Oral antibiotic	Total antibiotic course
1	Gram-positive cocci	*Staphylococcus aureus*/*epidermidis*	Vancomycin	6	Cefuroxime axetil	4 weeks (IV 6 days, followed by oral)
2	Gram-positive cocci	*Staphylococcus aureus*/*epidermidis*	Vancomycin	6	Cefuroxime axetil	4 weeks (IV 6 days, followed by oral)
3	Gram-positive cocci	*Staphylococcus aureus*/*epidermidis*	Vancomycin	9	Cefuroxime axetil	4 weeks (IV 9 days, followed by oral)
4	Gram-positive cocci	*Staphylococcus aureus*/*epidermidis*	Vancomycin	9	Cefuroxime axetil	4 weeks (IV 9 days, followed by oral)
5	Gram-positive cocci	*Staphylococcus aureus*/*epidermidis*	Vancomycin	6	Cefuroxime axetil	4 weeks (IV 6 days, followed by oral)
6	Gram-positive cocci	*Staphylococcus aureus*/*epidermidis*	Vancomycin	6	Cefuroxime axetil	4 weeks (IV 6 days, followed by oral)
7	Gram-positive cocci	*Staphylococcus aureus*/*epidermidis*	Vancomycin	9	Cefuroxime axetil	4 weeks (IV 9 days, followed by oral)
8	Gram-negative bacilli	Gram-negative bacillus	Meropenem	6	Levofloxacin	4 weeks (IV 6 days, followed by oral)

Follow-up: all eight patients were followed for 1 year post-discharge, with no instances of infection recurrence or readmission observed (see Table 7).

**Table 7:** Hospitalization and follow-up outcomes.

Patient ID	Hospital stay (days)	Suture removal time (Post-op day)	Secondary debridement	Infection control status	1-year follow-up outcome
1	9	8	No	Complete	No recurrence
2	10	8	No	Complete	No recurrence
3	9	8	No	Complete	No recurrence
4	11	8	No	Complete	No recurrence
5	9	8	No	Complete	No recurrence
6	10	8	No	Complete	No recurrence
7	9	8	No	Complete	No recurrence
8	11	8	No	Complete	No recurrence
Total/mean	9.75	8	0/8 (0%)	8/8 (100%)	0/8 recurrences

All eight SSI patients achieved complete clinical cure without recurrence during the 12-month post-treatment follow-up. None of the patients required hardware removal or staged reimplantation, confirming the efficacy of the combined debridement, irrigation, and antimicrobial therapy protocol. Post-treatment imaging and neurological assessments revealed no structural damage or neurological deficits related to infection or intervention, and all patients resumed regular DBS neuromodulation therapy with restored therapeutic benefits.

## 4 Discussion

The DBS surgical procedure is relatively complex, and surgeons change instruments significantly more frequently than in other neurosurgical operations. Factors such as the wide variety of instruments, high frequency of changes, and extensive operative field all increase the risk of infection. Although the operative time is relatively short, the intricate workflow elevates the probability of infection. Furthermore, the implantation of DBS-related devices (foreign bodies) significantly heightens the risk of surgical site infection (SSI).

Infection is the most common complication of DBS surgery, with reported rates ranging from approximately 2.6% to 10% (1). Deep brain stimulator infection not only interrupts therapeutic benefits but also significantly prolongs hospital stays and increases healthcare costs. Although multiple medical centers are exploring various strategies to reduce DBS hardware-related infection rates, there is still a lack of randomized controlled trial (RCT) evidence; existing studies are predominantly retrospective cohort analyses. Beyond systemic antibiotic prophylaxis, the literature reports the local application of vancomycin powder (VP) to the surgical incision for SSI prevention, with lower SSI rates observed in patients receiving VP (though the association between VP and reduced SSI risk did not fully reach the pre-specified level of statistical significance).

To the best of our knowledge, this study is the first to demonstrate that intraoperative wound irrigation with either hydrogen peroxide (H_2_O_2_) or povidone-iodine (PVP-I) solution reduces the risk of postoperative DBS infection by 80.4%, with both antiseptics showing comparable efficacy. Both disinfectants have a long history of use in surgery. Hydrogen peroxide, a versatile chemical agent, is widely employed across medical fields, particularly in plastic surgery, orthopedics, general surgery, and neurosurgery (13). Its neurosurgical application dates back to cerebellar abscess debridement in 1893. The antimicrobial mechanism of hydrogen peroxide relies on the Fenton reaction: decomposition of H_2_O_2_ generates oxygen free radicals, which cause oxidative stress damage to microbial DNA, proteins, and other cellular structures, ultimately leading to pathogen death (14). As a broad-spectrum antiseptic, it effectively inhibits the growth of bacteria, fungi, viruses, and yeasts (15).

However, hydrogen peroxide application carries potential complications, primarily neurotoxicity, impaired wound healing, and gas embolism. Its negative impact on wound healing is mediated through damage to keratinocytes and fibroblasts (16). The substantial production of oxygen free radicals explains its neurotoxicity. The most severe complication is gas embolism caused by wound irrigation, potentially leading to neurological sequelae, hemodynamic instability, subcutaneous emphysema, limb ischemia, or even death (17). Therefore, the core protective measure when using hydrogen peroxide is meticulous dural closure to prevent its entry into the cranial cavity, which could cause intracranial pneumatosis, neural injury, or gas embolism. To mitigate embolism risk, we ensured thorough wound hemostasis before irrigation and immediately flushed any residual hydrogen peroxide with saline after irrigation.

Sole reliance on hydrogen peroxide irrigation has antimicrobial limitations: certain bacteria (e.g., common skin pathogens *Staphylococcus aureus* and *Pseudomonas aeruginosa*) can secrete catalase, diminishing its bactericidal effect (18). Studies indicate that both hydrogen peroxide and povidone-iodine solutions effectively reduce SSI rates (19). Consequently, the other antiseptic agent selected for this study was povidone-iodine solution (PVP-I). As the most commonly used iodine preparation today, PVP-I is a stable complex of polyvinylpyrrolidone and elemental iodine. Free molecular iodine (I_2_) in the solution is its primary bactericidal component (20). I_2_ freely penetrates microbial cells and exerts its germicidal action through multiple mechanisms: blocking arginine/histidine hydrogen bond formation and binding to tyrosine phenolic groups; oxidizing sulfhydryl groups of cysteine/methionine, denaturing proteins; and binding to fatty acid carbon chains and nucleotides (adenine, cytosine, and guanine), disrupting nucleic acid structures and cell membrane integrity (21). I_2_ comprehensively damages microbial cell membranes, inhibits the respiratory chain, interferes with electron transport, blocks protein synthesis, and denatures nucleic acids. It is a broad-spectrum, non-resistance-inducing bactericide effective against bacteria, viruses, fungi, and protozoa (22). Thus, PVP-I is an ideal surgical antiseptic.

Local application of hydrogen peroxide or diluted povidone-iodine to the incision enables targeted infection control. This study is the first to demonstrate that intraoperative irrigation significantly reduces the risk of postoperative DBS infection by 80.4%, with no observed complications such as impaired wound healing, gas embolism, or neurological injury. This finding aligns with studies in orthopedic joint arthroplasty (23). However, combined use did not demonstrate a synergistic effect, potentially due to the chemical neutralization reaction where H_2_O_2_ oxidizes iodide ions to form hypoiodous acid, reducing bactericidal efficacy (15).

After confirming balanced baseline characteristics (age, sex, BMI, ASA classification, fasting blood glucose, etc.) with no statistically significant differences (*P* > 0.05) across the four patient groups, analysis of 385 DBS procedures revealed that antiseptic irrigation (H_2_O_2_, PVP-I, or their combination) significantly reduced SSI risk. The pooled intervention groups (^*^n^*^ = 290) had an infection rate of 1.03%, representing an absolute risk reduction (ARR) of 4.23% compared to the saline group (^*^n^*^ = 95, 5.26%; *P* = 0.039), and a relative risk reduction (RRR) of 80.4% (NNT = 24). Individual group comparisons did not reach significance (e.g., H_2_O_2_ group 1.04% vs. Saline group, *P* = 0.212), attributable to the rarity of infection events (total *n* = 8) and insufficient statistical power (32%) due to limited sample size per group (^*^n^*^ ≈ 96). By pooling intervention groups sharing the same “chemical antimicrobial mechanism,” the sample size increased to 290 (three infections), boosting power to 82%, thereby avoiding a Type II error and adhering to CONSORT guidelines. The highly consistent infection rates across intervention groups (1.03%−1.04%) support homogeneity, and the lack of difference between H_2_O_2_ and PVP-I groups (*P* > 0.99) confirms equivalence. The absence of synergy in the combination group (*P* > 0.05) may relate to overlapping antimicrobial spectra or limited sample size. Although constrained by its single-center design and relatively small sample size, the results provide Level III evidence for DBS infection prevention. Future multicenter RCTs are needed to optimize protocols and assess long-term electrode compatibility.

Pathogen spectrum analysis indicated predominant staphylococci in the saline group, consistent with typical DBS postoperative pathogens. The occurrence of an *Enterobacteriaceae* infection in the combination group (*n* = 1) hints at a possible mechanism whereby antiseptic irrigation disrupts skin flora balance, facilitating Gram-negative colonization. Given the small number of infections, this interpretation is speculative and warrants investigation in larger cohorts.

Management of SSIs showed that all eight infected cases successfully retained hardware while controlling infection. Key factors included: (1) Prompt intervention: early detection and management prevented infection spread; (2) Thorough debridement and sequential irrigation: necrotic tissue removal followed by sequential irrigation using the combined prophylaxis group method (100 mL H_2_O_2_ + 100 mL PVP-I sequentially) to eradicate residual pathogens; (3) Primary closure: avoiding drain placement to prevent secondary drain-related infections. While this approach demonstrated efficacy, its validation requires RCTs due to the limited sample size.

### 4.1 Regarding the surveillance period

The new NHSN standard defines the SSI surveillance period as 90 days postoperatively, but literature suggests this may underestimate the true incidence. Therefore, this study extended surveillance to 1 year. Seven of the eight SSIs occurred within 90 days, with one appearing at 353 days (nearly 1 year). As each intervention group had only one infection (H_2_O_2_, PVP-I, and Combination), median time estimates have limited representativeness. However, the late infection in the combination group (353 days) may suggest: (1) incomplete eradication of early biofilm by the antiseptic; (2) long latency period of low-virulence pathogens (e.g., *Enterobacteriaceae*); (3) the need for extended follow-up to monitor delayed infections. Given the small number of SSI cases, future studies with larger samples are needed to validate this trend.

### 4.2 Of particular note

The Enterobacter hormaechei detected in the combination group is a nosocomial multidrug-resistant organism. This suggests antiseptic irrigation might selectively suppress resident skin flora (e.g., staphylococci), enabling colonization by resistant Gram-negative bacteria. A similar phenomenon has been reported in cardiac pacemaker implantation. Subsequent research should investigate the impact of irrigation solutions on the local microbiome.

### 4.3 Study limitations

The single-center design limits generalizability; the effect of irrigation solution concentration gradients was not assessed; mechanisms of biofilm formation were not studied. Future multicenter trials are required to validate the optimal irrigation protocol, coupled with microbiome studies to delve deeper into infection prevention mechanisms.

### 4.4 Conclusion

This study demonstrates that single-agent irrigation with either hydrogen peroxide or povidone-iodine effectively reduces the risk of postoperative DBS infection. However, the potential risks of the combination regimen require further validation. Future large-scale, long-term studies are needed to optimize intraoperative infection control strategies, complemented by microbiological and molecular mechanistic exploration to advance precision infection prevention practices.

## Data Availability

The original contributions presented in the study are included in the article/Supplementary material, further inquiries can be directed to the corresponding author/s.
