# Supplementary material for: Preventive effect of intraoperative antiseptic irrigation on surgical site infections after deep brain stimulation: a retrospective cohort study
Source: Front Neurol. 2026 Sep 18;17:1883788. doi: 10.3389/fneur.2026.1883788 (PMC13632496; doi:10.3389/fneur.2026.1883788)
Supplement: Supplementary file 1 [file Table_1.docx]

| Supplementary Table 1: Comparison of Infection Site Distribution (Fisher's Exact Test) | | | | | |
| --- | --- | --- | --- | --- | --- |
| Infection Site | Saline Group (n=5) | Hydrogen Peroxide Group (n=1) | Povidone-Iodine Group (n=1) | Combination Group (n=1) | Total |
| Chest | 2 | 0 | 0 | 1 | 3 |
| Head (Electrode Entry) | 1 | 1 | 1 | 0 | 3 |
| Retroauricular | 2 | 0 | 0 | 0 | 2 |
| Total | 5 | 1 | 1 | 1 | 8 |
| (p=0.572) | | | | | |
